# A Low-Resources TDC for Multi-Channel Direct ToF Readout Based on a 28-nm FPGA

**DOI:** 10.3390/s21010308

**Published:** 2021-01-05

**Authors:** Mojtaba Parsakordasiabi, Ion Vornicu, Ángel Rodríguez-Vázquez, Ricardo Carmona-Galán

**Affiliations:** Instituto de Microelectrónica de Sevilla, IMSE-CNM (CSIC-Universidad de Sevilla), Avda. Américo Vespucio s/n, Parque Científico y Tecnológico de La Cartuja, 41092 Seville, Spain; mojtaba@imse-cnm.csic.es (M.P.); ivornicu@imse-cnm.csic.es (I.V.); angel@imse-cnm.csic.es (Á.R.-V.)

**Keywords:** field programmable gate array (FPGA), tapped-delay-line (TDL), thermometer-to-binary (T2B) encoder, multichannel TDCs, time-to-digital converter (TDC), time-of-flight (ToF), single-photon avalanche diode (SPAD)

## Abstract

In this paper, we present a proposed field programmable gate array (FPGA)-based time-to-digital converter (TDC) architecture to achieve high performance with low usage of resources. This TDC can be employed for multi-channel direct Time-of-Flight (ToF) applications. The proposed architecture consists of a synchronizing input stage, a tuned tapped delay line (TDL), a combinatory encoder of ones and zeros counters, and an online calibration stage. The experimental results of the TDC in an Artix-7 FPGA show a differential non-linearity (DNL) in the range of [−0.953, 1.185] LSB, and an integral non-linearity (INL) within [−2.750, 1.238] LSB. The measured LSB size and precision are 22.2 ps and 26.04 ps, respectively. Moreover, the proposed architecture requires low FPGA resources.

## 1. Introduction

Time-to-Digital Converters (TDCs) play a key role in a broad range of applications that require time measurement. One of the most relevant characteristics in three-dimensional (3D) imaging and ranging applications based on Single-Photon Avalanche Diode (SPAD) is the direct Time-of-Flight (ToF) capability [1]. To this end, high-resolution TDCs are highly demanded for 3D imaging [2,3], Fluorescence Lifetime Imaging Microscopy (FLIM) [4,5], and Positron Emission Tomography (PET) [6,7]. Furthermore, the increase in the number of detector modules and the requirement for real-time acquisition have led to the widespread utilization of multi-channel TDCs.

In recent years, Field-Programmable Gate Arrays (FPGAs) have been considered as an interesting implementation platform for fully-digital TDCs because of their flexibility, faster development phase, and lower implementation cost than Application-Specific Integrated Circuits (ASICs). Additionally, FPGA’s carry elements, whose intrinsic propagation delays can be used as a sort of fine time interpolator, have made FPGAs a suitable solution to implement high-resolution TDCs [8,9].

Different techniques for implementing TDCs on FPGA have been introduced in recent years [9,10], depending on the application’s specific requirements. Seeking to expand the measurable time interval and achieve higher time resolutions, the Nutt method, which combines a coarse counter and a time interpolator, is the most extended technique in FPGA-based TDCs [11,12]. There are different approaches in the literature to implement the time interpolator, such as Tapped Delay Lines (TDLs) [11,13], Vernier Delay Lines (VDLs) [14,15], multiple clock phases [16,17], delay-line loop-shrinking [18], and stochastic TDCs such as a matrix of counters [19].

As a straightforward time interpolator, a TDL [11,13] employs the carry elements of the FPGAs as delay elements. The intrinsic propagation delay of the delay elements determines the resolution. In VDLs [14,15], which employ more resources, the resolution is determined by the difference of the delays in two different chains of delay elements. With the increasing improvement of FPGAs manufacturing process, both of these methods can achieve a sub-hundred-picosecond resolution. Multiple-phase clock interpolators [16,17] use different clock phases of the reference clock to reach sub-clock resolutions. Since only a few different phases of the main clock are usually available, the best achievable resolution in this method is limited. Another time interpolator is based on the delay-line loop-shrinking technique [18]. It consists of two delay-line loops which are similar in architecture and delay cells but different in routing and placement. These differences determine the resolution in this method. The main weakness of this approach is that the dead-time depends on the length of the interval. It may not be an appropriate technique for applications that cover long time intervals. Finally, in the method based on a matrix of counters [19] as a stochastic TDC, the delay cells are the routing resources that are built of metal tracks and insensitive to the drift of FPGA core voltage and ambient temperature. Although this method can reach high resolution, it employs more resources than the others, and thus, this method is not a suitable time interpolator for multi-channel purposes. In addition, it uses a large area to build the routing paths.

There are also other time interpolation techniques with better performance, such as wave union TDC [20], multi-chain TDL [21], dual-phase TDL [22], and Ring-Oscillator-based (RO-based) multi-measurement TDL [23]. To improve the TDL resolution without additional delay lines and reduce the nonlinearity, wave union TDCs measure multiple transitions generated by wave union launchers. In multi-chain TDL TDCs, each channel has more than one TDL, and the output code of each channel is obtained by averaging all output codes of TDLs. Dual-phase TDL TDCs consider two TDLs for each channel, and each of the TDLs covers a half of the clock period. This interpolation method replaces a long delay line with two shorter delay lines to minimize the clock skew. RO-based multi-measurement TDL uses a ring oscillator to improve time resolution. These methods enhance the linearity of the TDC at the expense of high resources consumption and/or additional dead-time. For high-resolution multi-channel applications that require as few resources as possible for each channel while achieving sub-hundred-picosecond resolution, TDL is the best choice. Won and Lee [24] improved the linearity of the TDL in FPGAs by introducing a tuned sampling pattern that selects different outputs of the carry elements as the outputs of the delay line. In their proposed TDC, changing the sampling pattern requires more resources.

Another challenging block of an FPGA-based TDC is the thermometer-to-binary (T2B) encoder, which converts the delay line state to a binary code. Traditional encoding approaches generate the output code by finding the transition point in the delay line (“one-hot” binary encoder), but it can be severely affected by bubble errors. There are several online and offline techniques to minimize these errors, such as bubble-proof encoding [25], bin realignment [26], and stepped-up tree encoder [27]. Wang and Liu [28] used both of the bin realignment and bin decimation techniques to minimize the nonlinearity. To improve the linearity performance, Chen and Li [29] integrated several techniques such as sub-tapped delay line averaging, tap timing tests, a compensated histogram, and a mixed calibration method. All these techniques decrease the bubble problem at the cost of increased dead-time and/or higher resource utilization and/or LSB size degradation. Wang et al. [30] used a ones-counter encoder, which only counts the number of ones in the TDL and converts it to a binary number. A ones-counter encoder has the global ability to correct for bubbles because it does not depend on the tap sequence.

An important issue in FPGA-based TDCs is the non-uniformity of the delay elements from the carry chain, caused by process variation and mismatch. It is reflected in large Differential and Integral Nonlinearities (DNL and INL). Therefore, calibration becomes crucial for FPGA-based TDCs. The average delay method [31] and the bin-by-bin estimation approach [32] have been proposed for calibration. Although the former is a faster technique, the latter is better suited to FPGA-based TDL TDCs, because the sizes of the TDL delay elements have large differences. The bin-by-bin method is feasible by using a statistical estimation approach named code density test [33]. A table containing the measured bin widths of each delay cell can be stored in a Random-Access Memory (RAM) [34,35] to implement the online calibration. The bin widths are either fixed [34] during the time intervals measurements or updatable [35]. These online updatable calibration tables contemplate the ambient changes while measuring the time intervals. The bin widths are then updated at the cost of more logic resources and/or decreased conversion rates.

The input stage is another important block of the TDC. The input signal may be, on the one hand, noisy and, on the other hand, either longer or shorter than the required width. Hence, the pulse needs to be filtered and its width should be equalized before being injected into the TDL. Additionally, the input stage detects the input event and sends an enabling signal to the next blocks of the TDC to inform them about receiving a new input signal. Different mechanisms have been proposed for the input stage [36,37,38]. Most of them consider the input signal as the clock of the flip-flop (FF). Tontini et al. [38] proposed an input stage that is highly synchronized and requires only one extra flip-flop.

The architecture reported in this paper shows the following strengths:

- Calibration technique
  1. The selection of the best S-C combination improves linearity. By searching for the most uniform configuration of the delay line, the linearity improves without time resolution degradation and additional dead time and resource usage.
  2. The online calibration resulted from a code density test, improving accuracy even further.
- Compactness
  1. The synchronization module consists of only two FFs, efficiently shaping any input pulse;
  2. The ones-zeros encoder requires low resource usage; it features a mere 8-ns propagation time. Moreover, it is robust against bubble errors, without requiring any additional correction logic.
  3. Reference frequency optimization for short TDL. It is adapted to the FPGA speed grade. With this approach, we can implement 400 TDC channels at 125 Msamples/s.
- Full electrical characterization
  1. We have provided full electrical characterization, including power consumption and resource usage estimation. These parameters are important in portable systems for distance ranging applications based on direct ToF, which requires multiple parallel channels.

The rest of the paper is organized as follows. The proposed low-resource FPGA-based tuned-TDL TDC, which uses a combinatory encoder of the time interpolator outputs, is described in Section 2. The evaluation procedure, the characterization of the TDC performance, and the comparison to the state-of-the-art works are provided in Section 3. Finally, Section 4 summarizes and concludes the article.

## 2. TDC Architecture

**Figure 1:**
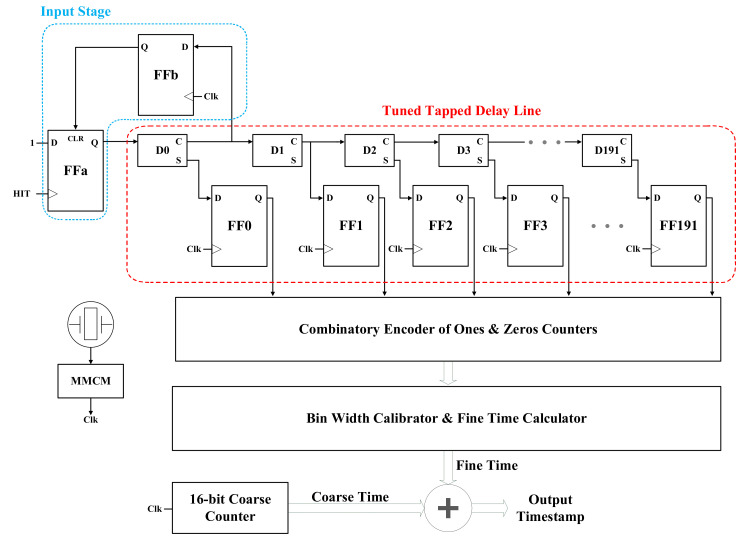
Architecture of the proposed FPGA-based TDC.

Figure 1 shows the architecture of the proposed FPGA-based TDC. It consists of an input stage, a coarse counter, a tuned-sampling-pattern TDL, a combinatory encoder of ones and zeros counters, and an online calibration block.

**Figure 2:**
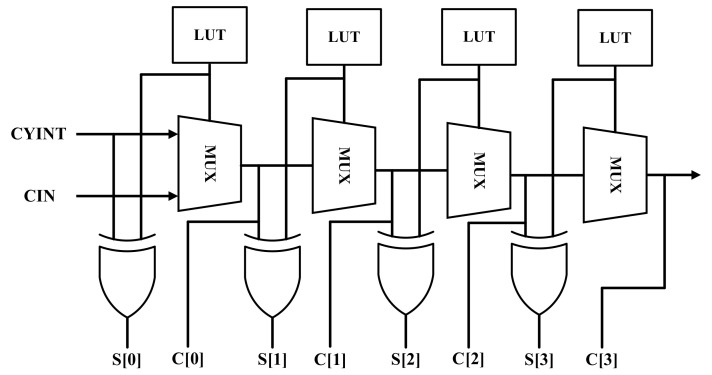
Simplified diagram of the structure of the CARRY4 block.

Modern FPGAs include Configurable Logic Blocks (CLB) which provide high-performance logic such as carry elements. A carry element is a dedicated high-speed component which is usually employed to implement fast arithmetic functions. The TDL in this paper employs cascade carry elements, each producing a short propagation delay. The implementation platform is Xilinx Artix-7 XC7A200T-1FBG484 (Xilinx Inc., San Jose, CA, USA), which is embedded in an Opal Kelly XEM7310 board (Opal Kelly Inc., Portland, OR, USA). The simplified structure of the CARRY4 block in these series-7 FPGAs is shown in Figure 2.

The input signal propagates through the multiplexers (MUX) and can be sampled at the carry out (C) or sum (S) nodes. The fine time resolution of TDL is determined by the propagation time through a delay unit. It depends on the FPGA fabrication technology, family, and speed grade. The linearity of the TDL is highly dependent on the sampling pattern, meaning the exact sequence of C’s and S’s is selected for sampling the output bits. Hence, to find the sampling pattern yielding a more linear TDC output, all possible sequences of C’s and S’s should be tested. Their nonlinearity metrics are then compared. To avoid further contributions to mismatch, all the delay elements of the TDL have to be located in the same clock region of FPGA. In this way, the clock skew is minimized. Moreover, the total delay of the line should be only slightly longer than the system clock period. In this design, a 250 MHz clock frequency and a TDL with 192 delay cells have been considered. According to the clock frequency and the number of coarse counter bits, the longest time interval covered is equal to 262.14 μs.

The input stage, shown in details in Figure 1, is used to properly shape the input signal. The first C of the delay line is employed to generate a clear signal of ‘FFa.’ When there is an input signal propagated through the delay line, the input stage resets the delay line in the next rising edge of the reference clock. Therefore, the width of the input signal equals the time interval between the input signal edge and the next rising edge of the clock. Furthermore, the input stage signals the next blocks about the incoming time sample injected into the TDL. Since the logic states of C and S are opposite, we cannot use the S instead of C in the input of the input stage. If the first element of the selected sampling pattern comes from C, the output of ‘FF0′ in Figure 1 can be used as the clear input of ‘FFa’ and ‘FFb’ can be removed from the circuit. However, as we will see later in the next section, the first bit of the selected sampling pattern comes from an S-type output.

The output of the TDL is a thermometer code that needs to be converted to a binary number. For that, we employ a thermometer-to-binary encoder (T2B) that needs to also take into account the TDL bubble errors. Ideally, the output of the TDL should be a clean thermometer code such as, for instance, 1111110000. However, because of uneven propagation delays within the TDL and the system clock skew, in practice, bubbles appear distorting the thermometer code. For instance, instead of 1111110000, the sampled state of the TDL could be 1101010000. This can lead to serious errors in the output binary code. Therefore, it is essential to design an encoder that avoids these kinds of errors. Moreover, since the TDL is tuned to a particular sampling pattern, and the states of each C and S pairs are opposite, two separate encoders would be required to find the transitions in both of them. Consequently, more resources are needed. To minimize the resource usage while suppressing bubbles in the thermometer code, we have introduced a combinatory ones and zeros counters encoder. In this case, we are counting the ones for C codes and zeros for S codes. This does not depend on the transition stage in the TDL, and therefore, the output is not severely affected by bubbles.

**Figure 3:**
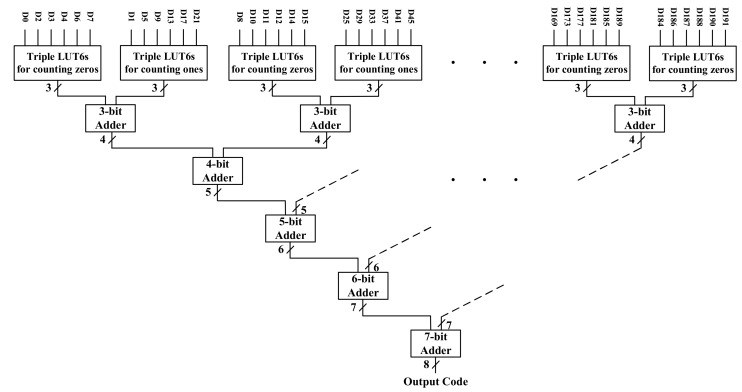
Combinatory encoder of ones and zeros counters.

The use of resources in this T2B encoder is equal to the case in which the same type of output is sampled for all of the delay elements. The architecture of the encoder is shown in Figure 3. In the first stage, the lookup tables (LUTs) connected to S nodes are configured to count the number of zeros, and the LUTs connected to C nodes are assigned to count the number of ones. In the next stages, the partial results have been combined to calculate the final binary number. Each set of LUTs consists of three 6-input LUTs and it converts 6-thermometer bins to a 3-bit binary code. A summary of the characteristics of the encoder is displayed in Table 1.

To implement real-time calibration, a table that maps each output binary code to a code representing the exact delay time has been built. The time related to each number has been obtained by a code density test which estimates the width of each bin. The time assignment procedure is as follows. Half of the first bin width corresponds to the delay mapped to the number ‘1.’ Half of the second bin width and the entire width of the first bin are added to reach the delay (from the origin) equivalent to the number ‘2.’ The procedure is the same for the other numbers and is summarized as follows:

(1) $$t_{k}=\frac{w_{k}}{2}+\overset{k-1}{\underset{i=0}{\sum }}w_{i}$$

where $t_{k}$ is the total delay (from the origin) related to number ‘k’ and $w_{k}$ is the measured bin width of the k-th bin.

Each binary number obtained from T2B is mapped into a new time interval by using the above equation. The resulted calibration table extracted from this equation and experimental measurements is shown and compared with the ideal transfer function in the next section.

## 3. Experimental Results

### 3.1. Measurements

**Figure 4:**
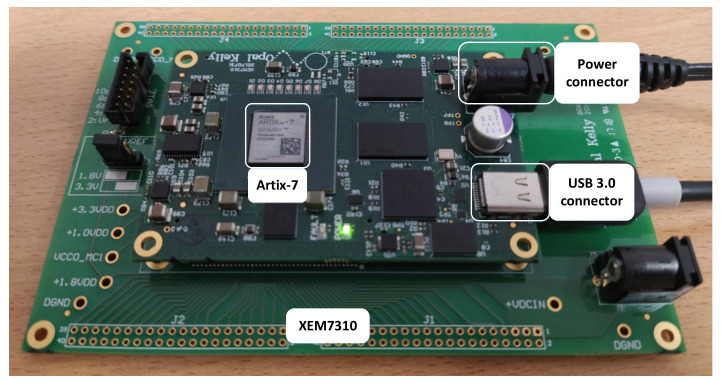
Evaluation board of the proposed TDC.

**Figure 5:**
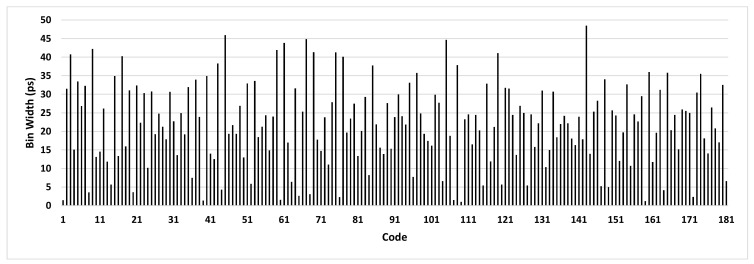
Measured bin widths of a TDC with “SCSS” sampling pattern resulted from code density test.

The proposed TDC has been implemented on the Artix-7 FPGA (XC7A200T-1FBG484) of an Opal Kelly XEM7310 board [39], as Figure 4 shows. To perform the code density test and send the results through the USB link, API components such as WireIn and PipeOut have been used [40]. The CFGMCLK output of the STARTUPE2 primitive has been used as the input source for the code density test [41]. Since this signal is generated by the internal ring oscillator of the ARTIX-7, there is no correlation between it and the system clock. We have collected 114,688 samples for the code density test. Then, we have measured the TDC bin widths with the following procedure. First, we have extracted the number of counts for each distinct sample. Then, this number is divided by the total number of samples. Finally, to estimate the bin width, the result is multiplied by the clock period of the TDC, i.e., 4000 ps in the proposed design. The bin widths of one of the TDCs are shown in Figure 5. This TDC employs a sampling pattern denoted by “SCSS,” where letters indicate the selected outputs of the CARRY4 delay cells, respectively. The reason for this choice will be explained later in this section.

FPGA-based TDCs have different sources of nonlinearity, such as clock skew, target device structure, local deviations of transistor characteristics, and ambient conditions. The effects of most of these sources can be minimized without using any additional resources by accounting for them during design and implementation.

First of all, all the delay elements of TDL have been placed in the same region to avoid clock region crossings. The clock skew between the regions can be a few hundreds of picoseconds, which can deteriorate the linearity of the TDC. Moreover, because of process variations, changing the position of the TDL in the same region also has an effect on the linearity. Therefore, the TDL has been placed in different columns of the same clock region and the results have been compared to find the best place for the TDL. Additionally, the position of the input stage has a direct relation with the linearity of the TDC. Thus, we have considered various positions for the input stage and compared their results.

Modern FPGAs, like Xilinx 7 series, contain a set of clocking resources, such as Mixed-Mode Clock Manager (MMCM), phase-locked loop, and different types of buffers [42]. To minimize the jitter of the system clock, an MMCM module is used. The TDC system is placed within a single clock region. We can use dedicated buffers, specifically designed for this kind of system. These buffers have access to high-speed, low skew local routing resources and can be driven by the MMCM.

To find out the most linear sampling pattern, the code density test has to be executed for all possible sampling patterns. To do this, we have designed and implemented different combinatory ones and zeros counters encoder for each of the sampling patterns. Then, we have tested all the patterns on the target device. Then, their DNL and INL values have been calculated as follows:

(2) $${\mathrm{DNL}}_{k}=\frac{w_{k}-w_{\mathrm{LSB}}}{w_{\mathrm{LSB}}}$$

(3) $${\mathrm{INL}}_{k}=\overset{k-1}{\underset{i=0}{\sum }}{\mathrm{DNL}}_{i}$$

where $w_{\mathrm{LSB}}$ is the LSB size and according to the measurements, is equal to 22.2 ps. Additionally, the number of active bins is 181.

**Figure 6:**
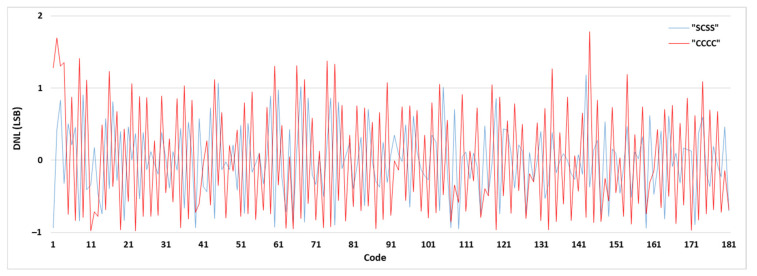
DNL results of “SCSS” and “CCCC” sampling patterns.

**Figure 7:**
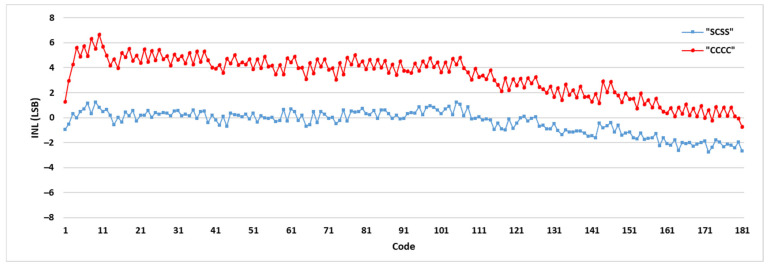
INL results of “SCSS” and “CCCC” sampling patterns.

**Figure 8:**
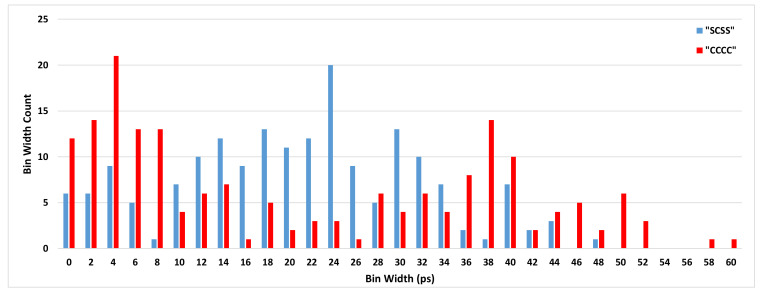
Bin width distributions of “SCSS” and “CCCC” sampling patterns.

To illustrate the effect of the sampling pattern on the TDC performance, the DNLs and INLs of some combinations are shown in Table 2. Among all the sampling patterns, “SCSS” has reached the most linear results and has been selected to be used in the final TDC system. The DNL and INL values of the selected sampling pattern (“SCSS”) have been compared with the ordinary sampling pattern (“CCCC”) in Figure 6 and Figure 7, respectively. Figure 8 shows the bin width distributions of “SCSS” and “CCCC” sampling patterns. These plots demonstrate the notable linearity improvement of the selected sampling pattern accompanied by the proposed encoder in the presented architecture.

**Figure 9:**
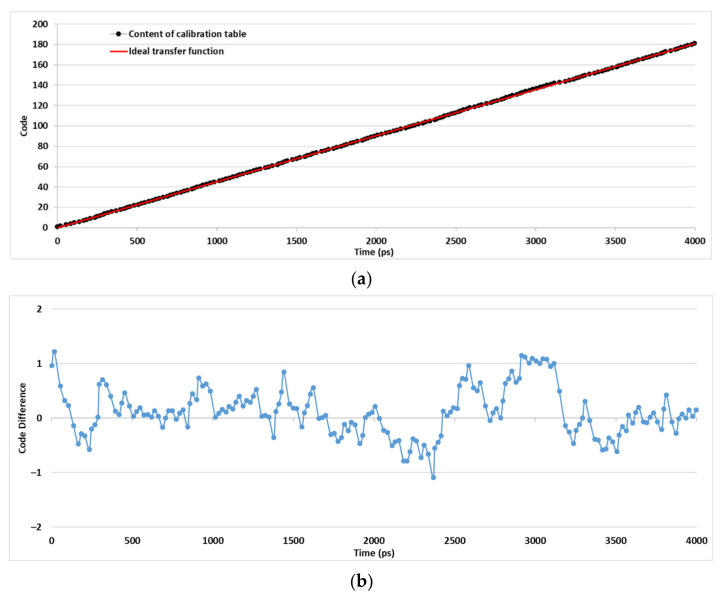
(**a**) Comparison of the TDC calibration table content and the ideal transfer function. (**b**) The differences between the codes.

Using the calibration method explained in Section 2, the calibration table of the proposed TDC has been calculated. In Figure 9, the content of the calibration table is compared with the ideal transfer function. We can compute the accuracy based on the measured and the ideal static characteristic. The absolute accuracy is the maximum deviation of the TDC calibration table from the ideal transfer function. The absolute accuracy of the proposed TDC is equal to 27.04 ps.

**Figure 10:**
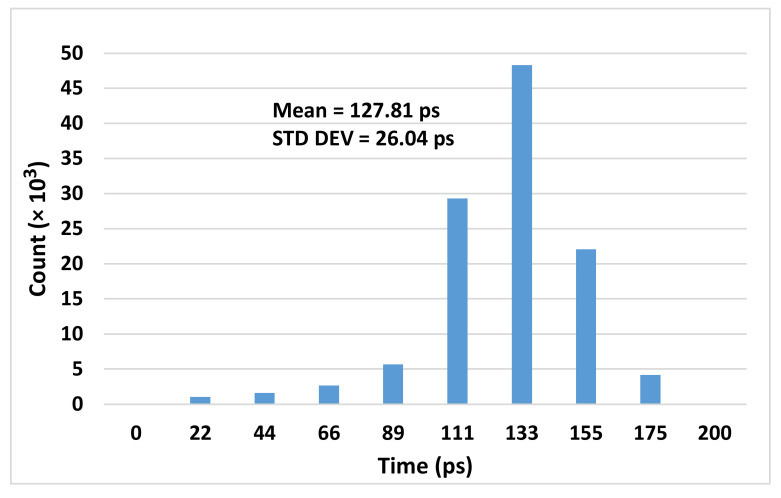
Measurement histogram of a constant time interval.

**Figure 11:**
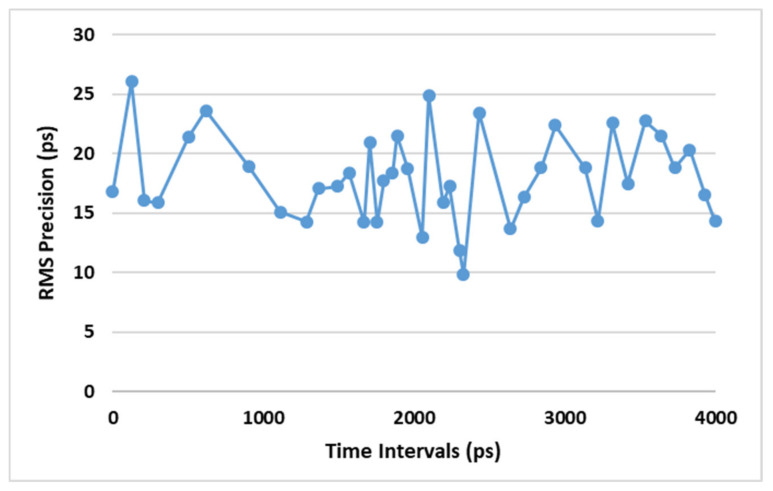
RMS precision of the different time intervals.

To evaluate the TDC measurement precision, a constant time interval has been measured by two TDC channels. One channel measures the start time of the interval and the other channel estimates its stop point. The time interval is calculated by subtracting the stop timestamp from the start one. Figure 10 shows the histogram of 114,688 samples measured by two TDC channels. The mean value and the standard deviation (STD DEV) of the time interval are 127.81 ps and 26.04 ps, respectively. We generated the different time intervals by hiring the IDELAY2 primitive of the FPGA. Since the time intervals are generated in the FPGA, they have less jitter than the intervals generated outside the FPGA. Moreover, an IDELAYCTRL calibrates the IDELAY2 to realize an accurate time interval. The RMS precision of the different time intervals are shown in Figure 11. Since the time interval has been fixed during the tests, the achieved standard deviation is the single-shot precision of the TDC. The standard deviation is calculated as follows:

(4) $$\sigma =\frac{1}{\sqrt{N-1}}\sqrt{\overset{N}{\underset{i=1}{\sum }}{\left(t_{i}-\frac{\sum_{j=1}^{N}t_{j}}{N}\right)}^{2}}$$

where σ is the standard deviation, $t_{i}$ is the result of i-th measurement, and N is the number of measurements.

**Figure 12:**
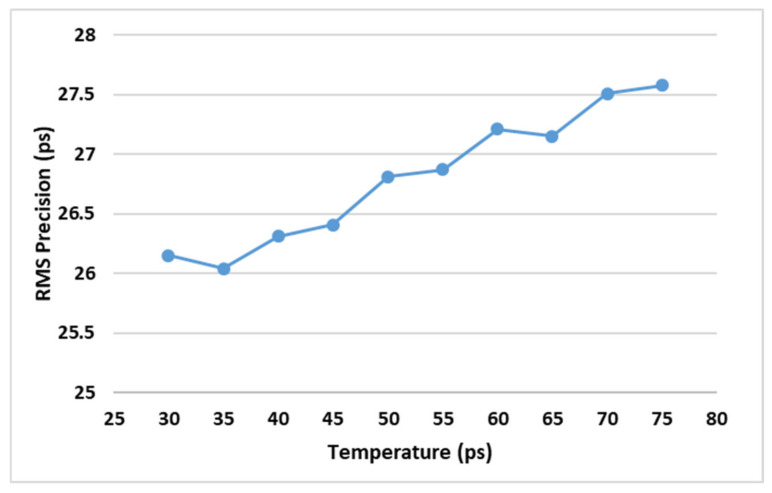
RMS precision variations with temperature.

To estimate the RMS precision variations over temperature, the code density test is carried out in different temperatures from 30 °C to 75 °C and the corresponding RMS precision variations are shown in Figure 12. Figure 12 indicates that the RMS precision degraded slightly with increasing temperature.

Table 3 shows data regarding the usage of logic resources and the power consumption of one TDC channel. These data are extracted for the implementation report and demonstrates the low resource utilization and the low power consumption of the proposed TDC. The characteristics of the proposed TDC are summarized in Table 4.

### 3.2. Comparison

**Figure 13:**
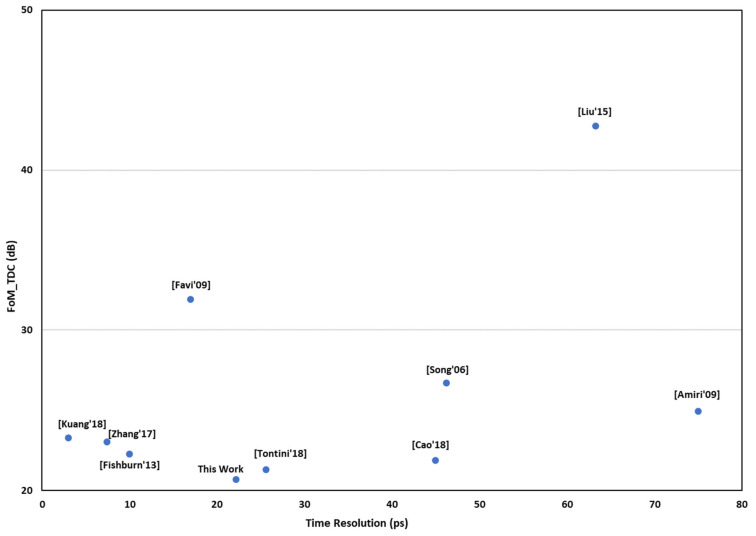
Comparison of FoM_TDC.

Table 5 provides a comparison with state-of-the-art FPGA-based TDCs. Note from the table that only some works report power consumption data, a few of them in detail and the others without indicating whether the reported amount is the total on-chip power consumption or not. Although the lack of data hinders comparison regarding power, the columns of the table highlight that the proposed TDC features low non-linearity and dead time while having a resource usage considerably lower than other works. Obviously, FPGA-based TDC performance depends on the FPGA fabrication technology. For example, the LSB width of TDL-based TDCs depends on the family, generation, and speed grade of the target device and newer technologies potentially lead to better performance. To elucidate the comparison with other works, we have used a simplified version of the Figure of Merit (FoM_TDC) presented in [43], which excludes power consumption because data regarding power are not reported in most of the FPGA-based TDCs:

(5) $$\mathrm{FoM}\_\mathrm{TDC}=10\times log_{10}\left(\frac{1}{2^{ENoB}\times F_{S}}\right)$$

(6) $$ENoB=N_{\mathrm{bits}}-\log_{2}\left(\mathrm{INL}+1\right)$$

where $F_{S}$ is the conversion rate of the TDC and ENoB is the effective number of bits. The FoM_TDC is plotted in Figure 13 for those references that report all the data required to calculate it.

## 4. Conclusions

We have designed and tested a novel FPGA-based TDC architecture delivering high performance with low resource usage. It has been implemented in an Artix-7 with a 250 MHz clock frequency. We have tested all the sampling patterns to find out the one rendering the highest linearity. We have employed a combinatory ones and zeros counters encoder to achieve high immunity to bubbles in the TDL, composed of different sampling elements with opposite logic states. The obtained resolution and single-shot precision are 22.2 ps and 26.04 ps, respectively. The measurement throughput is 125 MSa/s. The presented architecture can measure input intervals beyond 260 μs with 125 MSa/s conversion rate. The code density test results show [−0.953, 1.185] LSB DNL and [−2.750, 1.238] LSB INL. These characteristics make the proposed design suitable for a multi-channel direct ToF readout.

## Figures and Tables

**Table 1:** Summary of the ones and zeros combinatory counters encoder.

I/O		Resources		Processing Time
Input	Output	LUTs	FFs	
192 codes	8b	215	246	6 clocks

**Table 2:** Sampling patterns comparison.

Sampling Pattern	DNL (LSB)	INL (LSB)
CCCC	[−0.976, 1.779]	[−0.733, 6.660]
CSCS	[−0.987, 3.721]	[−0.733, 6.567]
SCSC	[−0.954, 1.425]	[−2.921, 1.274]
CCSC	[−0.978, 2.727]	[−0.119, 6.456]
CSCC	[−0.981, 3.698]	[−0.700, 6.700]
SCSS	[−0.953, 1.185]	[−2.750, 1.238]

**Table 3:** Resources usage and power consumption of one TDC channel.

Resource	Available	Utilization	Utilization (%)
LUT	133,800	216	0.16
FF	267,600	638	0.24
BRAM	365	2.50	0.68
Total Power Consumption	164 mW		
Dynamic Power	33 mW		

**Table 4:** Characteristics of the proposed TDC.

Parameter	Value/Range	Unit
Clock Frequency	250	MHz
Resolution	22.2	ps
Measurement Range	262.14	μs
Dead-Time	8	ns
Readout Speed	125	MSample/s
INL	[−0.953, 1.185]	LSB
DNL	[−2.750, 1.238]	LSB
Single-Shot Precision	26.04	ps

**Table 5:** Comparison with the state-of-the-art FPGA-based TDCs.

Ref.	Used Method	FPGA	LSB [ps]	Precision [ps]	DNL [LSB]	INL [LSB]	Dead-Time [ns]	Resources Usage	Power [mW]	ENoB	FOM_TDC (dB)
Song [11]	TDL	Virtex-2	46.2	65.8	1.10	2	10	NS	NS	5.42	26.71
Wu [20]	Wave Union	Cyclone II	30	25	NS	NS	5	NS	NS	NA	NA
Amiri [15]	Matrix of Vernier Delays	Spartan-3	75	300	2.5	3	4.17	NS	92	5	24.95
Favi [12]	TDL	Virtex-5	17	24.2	3.55	3	50	1208 Slices	NS	5	31.94
Buchele [17]	Multi-phase Clock	Virtex-5	160	68	0.8	NS	NS	NS	NS	NA	NA
Fishburn [13]	TDL	Virtex-6	10	19.6	1.5	2.25	3.3	NS	NS	5.30	22.29
Zhang [18]	Delay Line Loops Shrinking	SmartFusion	63.3	61.7	0.55	0.72	1410	NS	NS	6.22	42.77
Liu [21]	Multi-Meas. TDL	Kintex-7	9.4	9.5	4.6	NS	1.47	400 Slices	NS	NA	NA
Wang [28]	TDL + Bin Realignment & Decimation	Kintex-7	17.6	15	1	0.8	NS	NS	NS	7.15	NA
Won [22]	Dual-phase TDL + Online Cal.	Virtex-6	10	12.83	1.91	3.93	NS	NS	NS	5.70	NA
Cao [35]	TDL + Bin Realignment	Cyclone-IV	45	18	0.5	0.48	13.3	NS	NS	6.43	21.88
Wang [30]	Mul-Ch. TDL + ones Counter Encoder	Kintex-7	2.45	3.9	NS	NS	3.61	6258 FFs + 2433 LUTs	821	NA	NA
Zhang [19]	Matrix of Counters	Virtex-5	7.4	6.8	0.74	1.57	80	1265 Slices	1113	8.64	23.03
Kuang [23]	Multi-Meas. RO-based TDL	Kintex-7	3	5.76	NS	9	22	NS	NS	6.68	23.27
Chen [29]	sub-TDL + tap timing + histogram + mixed cal.	Virtex-7	10.54	14.59	0.08	0.11	NS	1916 FFs + 1145 LUTs	NS	7.85	NA
Tontini [38]	Input Stage + Tuned TDL	Spartan-6	25.6	37	1.23	2.96	8.69	415 Slices	131	6.01	21.29
This work	Input Stage+ Tuned TDL + Combinatory Encoder	Artix-7	22.2	26.04	1.18	2.75	8	638 FFs + 216 LUTs	164 (Total)	6.10	20.68

## Data Availability

The data presented in this study are available on request from the corresponding author. The data not contained in the article are not publicly available due to on-going result protection and technology transference processes.
